# An Efficient Algorithm to Count Tree-Like Graphs with a Given Number of Vertices and Self-Loops

**DOI:** 10.3390/e22090923

**Published:** 2020-08-22

**Authors:** Naveed Ahmed Azam, Aleksandar Shurbevski, Hiroshi Nagamochi

**Affiliations:** Department of Applied Mathematics and Physics, Kyoto University, Kyoto 606-8502, Japan; shurbevski@amp.i.kyoto-u.ac.jp (A.S.); nag@amp.i.kyoto-u.ac.jp (H.N.)

**Keywords:** trees, chemical graphs, enumeration, dynamic programming, polymer topology

## Abstract

Graph enumeration with given constraints is an interesting problem considered to be one of the fundamental problems in graph theory, with many applications in natural sciences and engineering such as bio-informatics and computational chemistry. For any two integers n≥1 and Δ≥0, we propose a method to count all non-isomorphic trees with *n* vertices, Δ self-loops, and no multi-edges based on dynamic programming. To achieve this goal, we count the number of non-isomorphic rooted trees with *n* vertices, Δ self-loops and no multi-edges, in $O(n^{2}\left(n+\Delta \left(n+\Delta \cdot \min\left\{n,\Delta \right\}\right)\right))$ time and $O(n^{2}\left(\Delta^{2}+1\right))$ space, since every tree can be uniquely viewed as a rooted tree by either regarding its unicentroid as the root, or in the case of bicentroid, by introducing a virtual vertex on the bicentroid and assuming the virtual vertex to be the root. By this result, we get a lower bound and an upper bound on the number of tree-like polymer topologies of chemical compounds with any “cycle rank”.

## 1. Introduction

Counting and generation of discrete objects are two fundamental problems in combinatorial mathematics and have many applications in the fields of natural science and engineering, such as computational chemistry and bioinformatics. The counting problem asks to count all possible objects under given constraints. On the other hand, the generation problem asks to list all possible objects under given constraints. One of the notable advantages of the counting problem is that we can know the size of the solution space before generating all solutions.

Different kinds of enumeration methods are used to solve counting and generation problems, where branching algorithms and Polya’s enumeration theorem are the two most commonly used methods for these problems. In branching algorithms, the computation is performed by following a computation tree, and the required solutions are attained at the leaves of the computation tree. It is important to mention that the branching algorithms can only count all solutions after generating each one of them, and therefore they are inefficient for the problem where we first want to know the size of the solution space before the generation of solutions.

The well-known Polya’s enumeration theorem [1,2] is used for counting all distinct objects. The idea of this method is to use the cyclic index of the group of symmetries of the underlying object to develop a generating function, which is then used to count all possible objects. Note that finding the group of symmetries and its cyclic index is a challenging task, which may make the use of Polya’s theorem harder for some problems.

The drawback of branching algorithms discussed above and the difficulty of using Polya’s theorem necessitate the exploration of new enumeration methods to solve counting problems efficiently. For an enumeration method, it is necessary to satisfy the following three conditions:

(i) Consider all solutions: The method does not miss any of the required objects;
(ii) Avoid duplication: The method does not count and generate isomorphic objects; and
(iii) Low computational complexity: The method can count and generate all solutions in low time and space complexity.

Designing such a method is not an easy task, because of the underlying symmetries and the computation difficulty for their detection.

Counting and generation of chemical compounds have a long history and numerous applications in designing novel drugs [3,4,5,6,7,8] and structure elucidation [9]. The problem of counting and generation of chemical compounds can be viewed as the problem of enumerating graphs with given constraints. There are several available chemical compound enumeration tools [10,11,12]. We can divide these tools into two classes. One class of enumeration tools treats general graph structures [10,12]. In the other class, the tools are focused on enumerating some restricted chemical compounds. One such tool is Enumol2 [11]. Enumeration of restricted chemical compounds with specialized tools is more efficient than with the tools which use general graph structures. This led to a new trend of developing efficient enumeration of restricted chemical compounds in the field of chemoinformatics [13].

**Figure 1:**
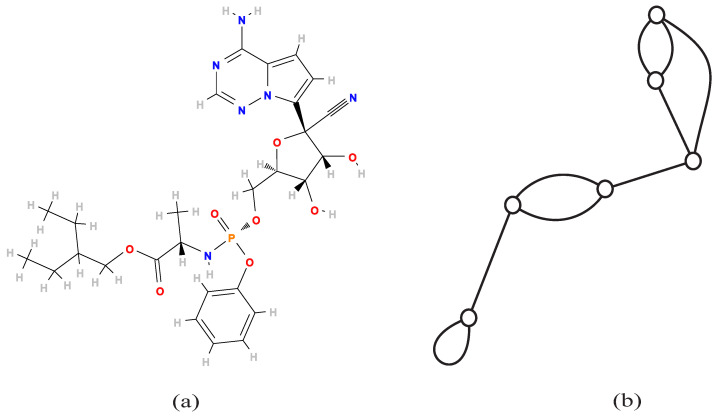
The chemical compound Remdesivir C${}_{27}$H${}_{35}$N${}_{6}$O${}_{8}$P and its polymer topology: (**a**) chemical structure of Remdesivir C${}_{27}$H${}_{35}$N${}_{6}$O${}_{8}$P obtained from the PubChem database; (**b**) the polymer topology of Remdesivir with six vertices, two multi-edges of multiplicity 2, one self-loop and cycle rank 4.

A polymer is a large molecule with interesting chemical properties consisting of many sub-molecules. From a graph-theoretic perspective, we represent the structure of a polymer with a graph *G* called *polymer topology*, possibly with self-loops and multi-edges, such that *G* is connected and the degree of each vertex in *G* is at least three [14]. For a chemical graph, we get its polymer topology by repeatedly removing the vertices of degree one and two. For example, the polymer topology of Remdesivir C${}_{27}$H${}_{35}$N${}_{6}$O${}_{8}$P Figure 1a, a potential candidate of treatment for COVID-19, is illustrated in Figure 1b.

Tezuka and Oike [15] pointed out that a classification of polymer topologies will lay a foundation for the elucidation of structural relationships between different macro-chemical molecules and their synthetic pathways. Different kinds of graph-theoretic approaches have been applied to classify and enumerate polymer topologies [16,17]. For a connected graph *G*, possibly with self-loops and multi-edges, the *cycle rank* is defined to be the number of edges that must be removed to get a simple spanning tree of *G*. Recently, Haruna et al. [14] proposed a method to enumerate all polymer topologies with cycle rank up to five.

Notice that trees with no multi-edges but with Δ≥0 self-loops have cycle rank Δ and include all polymer topologies with the said structure. Therefore, it is of interest to count and generate all trees with no multi-edges and a given number of vertices and self-loops.

We use dynamic programming (DP) to count all mutually non-isomorphic trees with *n* vertices, Δ self-loops and no multi-edges. The basic idea of DP is to partition the original problem into subproblems that satisfy some recursive relations, and the union of their solution sets is equal to the solution set of the original problem. Unlike branching algorithms and Polya’s theorem, the main advantage of using the DP is that we can count all non-isomorphic structures without their generation and calculation of their group of symmetries. As an application of our results, we get lower and upper bounds on the number of tree-like polymer topologies with self-loops of a given cycle rank.

The rest of the paper is organized as follows: Section 2 reviews some notions and results related to graph theory. Section 3 explains our tree counting method. Section 4 makes some concluding remarks.

## 2. Preliminaries

Throughout this draft, the term *graph* stands for an undirected graph with no multi-edges and possibly with self-loops unless stated otherwise. Let *G* be a graph. We denote an edge between two vertices *u* and *v* in *G* by uv(=vu). Let V(G) and E(G) denote the vertex set and edge set of *G*, respectively. Let s(G) denote the number of self-loops in *G*. For a vertex v∈V(G), we denote by s(v) the number of self-loops on the vertex *v*. For a vertex *v* in *G*, let $N_{G}\left(v\right)$ denote the set of vertices incident to *v* except *v* itself and the *degree* deg${}_{G}\left(v\right)$ of *v* in *G* is defined to be $|N_{G}\left(v\right)|$. A graph *H* with the properties V(H)⊆V(G) and E(G)⊆E(G) is called a *subgraph* of *G*. A *simple path* between two distinct vertices u,v∈V(G) is defined to be a subgraph *P* of *G* with vertex set $V\left(P\right)=\left\{u=w_{1},w_{2},\ldots ,v=w_{k}\right\}$ and edge set $E\left(P\right)=\left\{w_{i}w_{i+1}|1\leq i\leq k-1\right\}$. A graph is called a *connected graph* if there is a path between any two distinct vertices in the graph. A *connected component* of a graph *G* is defined to be a maximal connected subgraph *H* of *G*, i.e., for any vertex v∈V(G)\V(H) it holds that every subgraph with the vertex set V(H)∪{v} is disconnected.

By Jordan [18], any simple tree with n≥1 vertices has either a unique vertex or edge, the removal of which creates connected components with at most ⌊(n−1)/2⌋ or exactly n/2 vertices, respectively. Such a vertex is called the *unicentroid*, the edge is called the *bicentroid*, and collectively they are called the *centroid* of the tree. It is important to note that there exits a bicentroid only for trees with an even number of vertices. A tree with a fixed vertex *r* is called a *rooted tree* with root *r*. Note that any tree can be uniquely viewed as a rooted tree by either regarding its unicentroid as the root, or in the case of a bicentroid, by introducing a virtual vertex on the bicentroid and assuming the virtual vertex as the root.

Let *H* be a rooted tree. Let $r_{H}$ denote the root of *H*. For any two distinct vertices u,v∈V(H), let $P_{H}\left(u,v\right)$ denote the unique simple path between them in *H*. For a vertex $v\in V\left(H\right)\backslash \left\{r_{H}\right\}$, we define the *ancestors* of *v* to be the vertices on the path $P_{H}\left(v,r_{H}\right)$ other than *v*. If *u* is an ancestor of *v*, then we call *v* a *descendant* of *u*. For a vertex $v\in V\left(H\right)\backslash \left\{r_{H}\right\}$, the *parent* p(v) of *v* is defined to be the ancestor *u* of *v* such that $u\in N_{H}\left(v\right)$. We call the vertex *v* a *child* of p(v). Two vertices with the same parent in *H* are called *siblings*. For a vertex v∈V(H), let $H_{v}$ denote the subtree of *H* rooted at *v* induced by *v* and its descendants.

Two rooted trees *T* and *H* are called *isomorphic* if there exists a bijection σ:V(T)→V(H) such that

(i) $\sigma \left(r_{T}\right)=r_{H}$;
(ii) for each vertex v∈V(T), it holds that s(v)=s(σ(v)); and
(iii) for any two vertices u,v∈V(T), it holds that uv∈E(T) if and only if σ(u)σ(v)∈E(H).

For any two integers n≥1 and Δ≥0, let H(n,Δ) denote a maximal set of mutually non-isomorphic rooted trees with *n* vertices and Δ self-loops, and we define $h\left(n,\Delta \right)≜\left|H(n,\Delta )\right|$.

## 3. Counting Tree-Like Graphs with a Given Number of Vertices and Self-Loops

We develop a method to compute for any two integers n≥1 and Δ≥0, the size h(n,Δ) of a maximal set H(n,Δ) of mutually non-isomorphic rooted trees with *n* vertices and Δ self-loops; i.e., we are interested in the following problem:

**Counting Problem**

**Input:** Two integers n≥1 and Δ≥0.

**Output:** h(n,Δ).

We solve this problem by using dynamic programming based on the information of the number of vertices and self-loops in the subtrees rooted at the children of the root of each tree in H(n,Δ). We define the following notions.

Let n≥1 and Δ≥0 be any two integers. For each tree H∈H(n,Δ), we define

$$\begin{array}{cc} {\mathrm{Max}}_{v}\left(H\right)≜ & \max\{\{|V\left(H_{v}\right)||v\in N_{H}\left(r_{H}\right)\}\cup \left\{0\right\}\},\\ {\mathrm{Max}}_{s}\left(H\right)≜ & \max\{\{s\left(H_{v}\right)|v\in N_{H}\left(r_{H}\right),|V\left(H_{v}\right)|={\mathrm{Max}}_{v}\left(H\right)\}\cup \left\{0\right\}\}. \end{array}$$

Note that for any tree H∈H(1,Δ), it holds that ${\mathrm{Max}}_{v}\left(H\right)=0$ and ${\mathrm{Max}}_{s}\left(H\right)=0$.

Let m,d≥0 be any two integers. We define

$$H\left(n,\Delta ,m_{\leq },d_{\leq }\right)≜\left\{H\in H\left(n,\Delta \right)|{\mathrm{Max}}_{v}\left(H\right)\leq m,{\mathrm{Max}}_{s}\left(H\right)\leq d\right\}.$$

Observe that by the definition of $H(n,\Delta ,m_{\leq },d_{\leq })$ it holds that

(i) $H\left(n,\Delta ,m_{\leq },d_{\leq }\right)=H\left(n,\Delta ,n-1_{\leq },d_{\leq }\right)$ if m≥n;
(ii) $H\left(n,\Delta ,m_{\leq },d_{\leq }\right)=H\left(n,\Delta ,m_{\leq },\Delta_{\leq }\right)$ if d≥Δ+1; and
(iii) $H\left(n,\Delta \right)=H\left(n,\Delta ,n-1_{\leq },\Delta_{\leq }\right)$.

Therefore, from now on, we assume that m≤n−1 and d≤Δ. Further, by the definition of $H(n,\Delta ,m_{\leq },d_{\leq })$ it holds that $H(n,\Delta ,m_{\leq },d_{\leq })\neq \emptyset$ (resp., $H(n,\Delta ,m_{\leq },d_{\leq })=\emptyset$) if “n=1” or “n−1≥m≥1” (resp., otherwise (n≥2 and m=0)).

We define

$$H\left(n,\Delta ,m_{=},d_{\leq }\right)≜\left\{H\in H\left(n,\Delta ,m_{\leq },d_{\leq }\right)|{\mathrm{Max}}_{v}\left(H\right)=m\right\}.$$

It follows from the definition of $H(n,\Delta ,m_{=},d_{\leq })$ that $H(n,\Delta ,m_{=},d_{\leq })\neq \emptyset$ (resp., $H(n,\Delta ,m_{=},d_{\leq })=\emptyset$) if “n=1” or “n−1≥m≥1” (resp., otherwise (n≥2 and m=0)). Further we have the following relation:

(1) $$\begin{array}{cc} \; & H\left(n,\Delta ,m_{\leq },d_{\leq }\right)=H\left(n,\Delta ,0_{=},d_{\leq }\right)\;\mathrm{if}\;m=0, \end{array}$$

(2) $$\begin{array}{cc} \; & H\left(n,\Delta ,m_{\leq },d_{\leq }\right)=H\left(n,\Delta ,m-1_{\leq },d_{\leq }\right)\cup H\left(n,\Delta ,m_{=},d_{\leq }\right)\;\mathrm{if}\;m\geq 1, \end{array}$$

where $H\left(n,\Delta ,m-1_{\leq },d_{\leq }\right)\cap H\left(n,\Delta ,m_{=},d_{\leq }\right)=\emptyset$ for m≥1.

Next we define

$$H\left(n,\Delta ,m_{=},d_{=}\right)≜\left\{H\in H\left(n,\Delta ,m_{=},d_{\leq }\right)|{\mathrm{Max}}_{s}\left(H\right)=d\right\}.$$

Note that if “n=1 and d=0” or “n−1≥m≥1” (resp., otherwise (“n=1 and d≥1” or “n≥2 and m=0”)), then by the definition of $H(n,\Delta ,m_{=},d_{=})$ it holds that $H(n,\Delta ,m_{=},d_{=})\neq \emptyset$ (resp., $H(n,\Delta ,m_{=},d_{=})=\emptyset$). Furthermore, we get the following relation for $H(n,\Delta ,m_{=},d_{\leq })$:

(3) $$\begin{array}{cc} \; & H\left(n,\Delta ,m_{=},d_{\leq }\right)=H\left(n,\Delta ,m_{=},0_{=}\right)\;\mathrm{if}\;d=0, \end{array}$$

(4) $$\begin{array}{cc} \; & H\left(n,\Delta ,m_{=},d_{\leq }\right)=H\left(n,\Delta ,m_{=},d-1_{\leq }\right)\cup H\left(n,\Delta ,m_{=},d_{=}\right)\;\mathrm{if}\;d\geq 1, \end{array}$$

where $H\left(n,\Delta ,m_{=},d-1_{\leq }\right)\cap H\left(n,\Delta ,m_{=},d_{=}\right)=\emptyset$ for d≥1.

Let n−1≥m≥0, and Δ≥d≥0 be four integers. Let $h(n,\Delta ,m_{\leq },d_{\leq })$, $h(n,\Delta ,m_{=},d_{\leq })$ and $h(n,\Delta ,m_{=},d_{=})$ denote the number of elements in the families $H(n,\Delta ,m_{\leq },d_{\leq })$, $H(n,\Delta ,m_{=},d_{\leq })$ and $H(n,\Delta ,m_{=},d_{=})$, respectively. We discuss recursive relations for $h(n,\Delta ,m_{\leq },d_{\leq })$ and $h(n,\Delta ,m_{=},d_{\leq })$ in Lemma 1.

**Lemma** **1.** *For any four integers n−1≥m≥0, and Δ≥d≥0, it holds that*

(i) *$h\left(n,\Delta ,m_{\leq },d_{\leq }\right)=h\left(n,\Delta ,0_{=},d_{\leq }\right)$ if m=0;*
(ii) *$h\left(n,\Delta ,m_{\leq },d_{\leq }\right)=h\left(n,\Delta ,m-1_{\leq },d_{\leq }\right)+h\left(n,\Delta ,m_{=},d_{\leq }\right)$ if m≥1;*
(iii) *$h\left(n,\Delta ,m_{=},d_{\leq }\right)=h\left(n,\Delta ,m_{=},0_{=}\right)$ if d=0; and*
(iv) *$h\left(n,\Delta ,m_{=},d_{\leq }\right)=h\left(n,\Delta ,m_{=},d-1_{\leq }\right)+h\left(n,\Delta ,m_{=},d_{=}\right)$ if d≥1.*

**Proof.**  The case (i) follows by Equation (1). The case (ii) follows by Equation (2) and the fact that for m≥1 it holds that $H\left(n,\Delta ,m-1_{\leq },d_{\leq }\right)\cap H\left(n,\Delta ,m_{=},d_{\leq }\right)=\emptyset$. By Equation (3) the case (iii) follows. The case (iv) follows by Equation (4) and the fact that for d≥1 it holds that $H\left(n,\Delta ,m_{=},d-1_{\leq }\right)\cap H\left(n,\Delta ,m_{=},d_{=}\right)=\emptyset$. □

Next we discuss some boundary conditions for our DP to compute h(n,Δ).

**Lemma** **2.** *For any four integers n−1≥m≥0, and Δ≥d≥0, it holds that*

(i) *$h(n,\Delta ,0_{=},d_{=})=1$ (resp., $h(n,\Delta ,0_{=},d_{=})=0$) if n=1 and d=0 (resp., otherwise (“n=1 and d≥1” or “ n≥2”));*
(ii) *$h\left(n,\Delta ,0_{=},d_{\leq }\right)=h\left(n,\Delta ,0_{\leq },d_{\leq }\right)=1$ (resp., $h\left(n,\Delta ,0_{=},d_{\leq }\right)=h\left(n,\Delta ,0_{\leq },d_{\leq }\right)=\;0$) if n=1 (resp., otherwise (n≥2));*
(iii) *$h(n,\Delta ,1_{=},d_{=})=1$ if “ n=2” or “ n≥3 and d=0”; and*
(iv) *$h\left(n,\Delta ,1_{=},d_{\leq }\right)=h\left(n,\Delta ,1_{\leq },d_{\leq }\right)=d+1$ if “ n=2” or “ n≥3 and d=0”.*

**Proof.** 

(i) The result follows from the definition of $H(n,\Delta ,0_{=},d_{=})$, since a tree *H* with max${}_{v}\left(H\right)=0$ exists if and only if |V(H)|=1 and max${}_{s}\left(H\right)=0$.
(ii) By Lemma 1(i), (ii) and (iv) it holds that $h\left(n,\Delta ,0_{\leq },d_{\leq }\right)=h\left(n,\Delta ,0_{=},d_{\leq }\right)=\sum_{p=0}^{d}h\left(n,\Delta ,0_{=},p_{=}\right)$. This and Lemma 2(i) imply the required result.
(iii) When n≥2, then for any tree $H\in H(n,\Delta ,1_{=},d_{=})$ it holds that $|N_{H}\left(r_{H}\right)|=n-1$. Thus for each $v\in N_{H}\left(r_{H}\right)$ it holds that $|V(H_{v})|=1$ and $s(H_{v})=d$ if “n=2” or “n≥3 and d=0”, i.e., $H_{v}\in H\left(1,d,0_{\leq },d_{\leq }\right)$. But by Lemma 2(ii) it holds that $h(1,d,0_{\leq },d_{\leq })=1$. Hence we have the required result.
(iv) Let “n=2” or “n≥3 and d=0”. By Lemma 1(iii) and (iv) it holds that $h\left(n,\Delta ,1_{=},d_{\leq }\right)=\sum_{p=0}^{d}h\left(n,\Delta ,1_{=},p_{=}\right)$. This and Lemma 2(iii) imply that
  (5) $$h(n,\Delta ,1_{=},d_{\leq })=d+1.$$
  Furthermore, by Lemma 1(iii) it holds that $h\left(n,\Delta ,1_{\leq },d_{\leq }\right)=h\left(n,\Delta ,0_{=},d_{\leq }\right)+h\left(n,\Delta ,1_{=},d_{\leq }\right)$. By Lemma 2(ii), we have $h\left(n,\Delta ,1_{\leq },d_{\leq }\right)=h\left(n,\Delta ,1_{=},d_{\leq }\right)$. Hence the result follows by Equation (5).

□

By Lemma 2, we can get that h(1,Δ)=1 and h(2,Δ)=Δ+1. Furthermore, Lemma 1(i)–(iv) give recursive relations for $h(n,\Delta ,m_{\leq },d_{\leq })$ and $h(n,\Delta ,m_{=},d_{\leq })$ which depend on $h(n,\Delta ,m_{=},d_{=})$. Thus for n≥3, m≥1, and Δ≥d≥0, our next goal is to develop a recursive relation for $h(n,\Delta ,m_{=},d_{=})$. For any tree $H\in H(n,\Delta ,m_{=},d_{=})$ and any vertex $v\in N_{H}\left(r_{H}\right)$, the subtree $H_{v}$ of *H* satisfies exactly one of the following three conditions:

(C-1) $\left|V(H_{v})\right|=m$ and $s(H_{v})=d.$
(C-2) $\left|V(H_{v})\right|=m$ and $0\leq s(H_{v})<d.$
(C-3) $\left|V(H_{v})\right|<m$ and $0\leq s(H_{v})\leq \Delta .$

**Figure 2:**
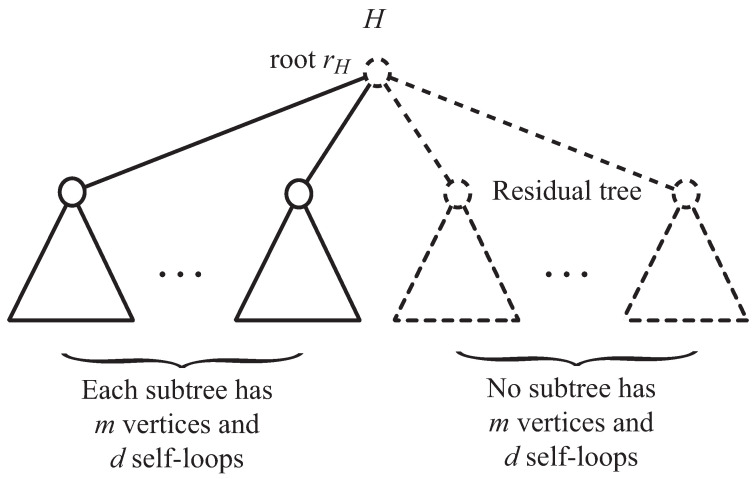
An illustration of a residual tree, where $H\in H(n,\Delta ,m_{=},d_{=})$ and the residual tree of *H* is shown by dashed lines.

For any tree $H\in H(n,\Delta ,m_{=},d_{=})$, we define the *residual tree* of *H* to be the subtree of *H* rooted at $r_{H}$ induced by the vertices $\underset{\begin{array}{c} v\in N_{H}\left(r_{H}\right),\\ H_{v}\in H\left(m,d,m-1_{\leq },d_{\leq }\right) \end{array}}{V\left(H\right)\backslash ⋃V\left(H_{v}\right)}.$ Note that the residual tree of a tree *H* has at least one vertex, i.e., the root of *H*. We give an illustration of a residual tree in Figure 2.

**Lemma** **3.** *For any four integers n≥3, m≥1, and Δ≥d≥0, and a tree $H\in H(n,\Delta ,m_{=},d_{=}),$ let $q=|\{v\in N_{H}\left(r_{H}\right)|H_{v}\in H\left(m,d,m-1_{\leq },d_{\leq }\right)\}|$. Then it holds that*

(i) *1≤q≤⌊(n−1)/m⌋ with q≤⌊Δ/d⌋ when d≥1.*
(ii) *The residual tree of H belongs to exactly one of the families $H(n\;-\;qm,\;\Delta \;-\;dq,m_{=},\min\{\Delta \;-\;dq,$${d\;-\;1\}}_{\leq })$ and $H(n-qm,\Delta -dq,\min{\left\{n-qm-1,m-1\right\}}_{\leq },\Delta -dq_{\leq })$.*

**Proof.** 

(i) Since $H\in H(n,\Delta ,m_{=},d_{=})$, there exists at least one vertex $v\in N_{H}\left(r_{H}\right)$ such that $H_{v}\in H\left(m,d,m-1_{\leq },d_{\leq }\right)$. This implies that q≥1. Also, it holds that n−1≥mq and Δ≥dq. This implies that q≤⌊(n−1)/m⌋ with q≤⌊Δ/d⌋ when d≥1.
(ii) Let *K* denote the residual tree of *H*. By the definition of *K* it holds that $K\in H(n-mq,\Delta -dq,n-mq-1_{\leq },\Delta -dq_{\leq })$. Furthermore, for each vertex $v\in N_{H}\left(r_{H}\right)\cap V\left(K\right),$ the tree $H_{v}$ satisfies exactly one of the conditions (C-2) and (C-3). Now, if there exists a vertex $v\in N_{H}\left(r_{H}\right)\cap V\left(K\right)$ such that $H_{v}$ satisfies condition (C-2), then d−1≥0, and hence $K\in H(n-qm,\Delta -dq,m_{=},\min{\left\{\Delta -dq,d-1\right\}}_{\leq })$. On the other hand, if condition (C-2) does not hold for any $v\in N_{H}\left(r_{H}\right)\cap V\left(K\right)$; i.e., either $N_{H}\left(r_{H}\right)\cap V\left(K\right)=\emptyset$ or for each $v\in N_{H}\left(r_{H}\right)\cap V\left(K\right)$ it holds that $|V\left(H_{v}\right)|\leq \min\left\{n-qm-1,m-1\right\}$ and $0\leq s(H_{v})\leq \Delta -dq$, then by the definition of *K* it holds that $K\in H(n-qm,\Delta -dq,\min{\left\{n-qm-1,m-1\right\}}_{\leq },\Delta -dq_{\leq }).$ This completes the proof.

□

For any five integers n≥3, m≥1, Δ≥d≥0, and t≥0, let c(m,d;t)≜h(m,d,m−1≤,d≤)+t−1t denote the number of combinations with repetition of *t* trees from the family $H(m,d,m-1_{\leq },d_{\leq })$. In Lemma 4, we give a recursive relation for $h(n,\Delta ,m_{=},d_{=})$.

**Lemma** **4.** *For any five integers n≥3, m≥1, Δ≥d≥0, and q, such that 1≤q≤⌊(n−1)/m⌋ with q≤⌊Δ/d⌋ when d≥1, it holds that*

(i) *$h\left(n,\Delta ,m_{=},d_{=}\right)=\sum_{q}c\left(m,d;q\right)h\left(n-qm,\Delta ,\min{\left\{n-qm-1,m-1\right\}}_{\leq },\Delta_{\leq }\right)$ if d=0;*
(ii) *$h\left(n,\Delta ,m_{=},d_{=}\right)=\sum_{q}c\left(m,d;q\right)\left(h\left(n-qm,\Delta -dq,m_{=},\min{\left\{\Delta -dq,d-1\right\}}_{\leq }\right)+h\left(n-qm,\Delta -dq,\min{\left\{n-qm-1,m-1\right\}}_{\leq },\Delta -dq_{\leq }\right)\right)$ if d≥1;*
(iii) *$h\left(n,\Delta ,m_{=},d_{=}\right)=\sum_{q}c\left(m,d;q-1\right)\left(\left(h\left(m,d,m-1_{\leq },d_{\leq }\right)+q-1\right)/q\right)h\left(n-qm,\Delta ,\min{\left\{n-qm-1,m-1\right\}}_{\leq },\Delta_{\leq }\right)$ if d=0; and*
(iv) *$h\left(n,\Delta ,m_{=},d_{=}\right)=\sum_{q}c\left(m,d;q-1\right)\left(\left(h\left(m,d,m-1_{\leq },d_{\leq }\right)+q-1\right)/q\right)\left(h\left(n-qm,\Delta -dq,m_{=},\min{\left\{\Delta -dq,d-1\right\}}_{\leq }\right)+h\left(n-qm,\Delta -dq,\min{\left\{n-qm-1,m-1\right\}}_{\leq },\Delta -dq_{\leq }\right)\right)$ if d≥1.*

**Proof.**  Let *H* be a tree in the family $H(n,\Delta ,m_{=},d_{=})$. By Lemma 3(i), there exists a unique integer *q*, 1≤q≤⌊(n−1)/m⌋ with q≤⌊Δ/d⌋ when d≥1, such that there are exactly *q* subtrees $H_{v}$ with $v\in N_{H}\left(r_{H}\right)$ and $H_{v}\in H\left(m,d,m-1_{\leq },d_{\leq }\right)$. Further, by Lemma 3(ii) the residual tree of *H* belongs to the family $H(n-qm,\Delta ,\min{\left\{n-qm-1,m-1\right\}}_{\leq },\Delta_{\leq })$ (resp., $H\left(n-qm,\Delta -dq,m_{=},\min{\left\{\Delta -dq,d-1\right\}}_{\leq }\right)\cup H\left(n-qm,\Delta -dq,\min{\left\{n-qm-1,m-1\right\}}_{\leq },\Delta -dq_{\leq }\right))$ if d=0 (resp., otherwise). Note that $H\left(n-qm,\Delta -dq,m_{=},\min{\left\{\Delta -dq,d-1\right\}}_{\leq }\right)\cap H\left(n-qm,\Delta -dq,\min{\left\{n-qm-1,m-1\right\}}_{\leq },\Delta -dq_{\leq }\right)=\emptyset$. This implies that for a fixed integer *q* in the range given in the lemma, the number of trees *K* in the family $H(n,\Delta ,m_{=},d_{=})$ with exactly *q* subtrees $K_{v}\in H\left(m,d,m-1_{\leq },d_{\leq }\right)$, for $v\in N_{K}\left(r_{K}\right)$, are

(a) $c\left(m,d;q\right)h(n-qm,\Delta ,\min{\left\{n-qm-1,m-1\right\}}_{\leq },\Delta_{\leq })$ if d=0; and
(b) $c\left(m,d;q\right)(h\left(n-qm,\Delta -dq,m_{=},\min{\left\{\Delta -dq,d-1\right\}}_{\leq }\right)+h\left(n-qm,\Delta -dq,\min{\left\{n-qm-1,m-1\right\}}_{\leq },\Delta -dq_{\leq }\right))$ if d≥1.

Note that, for m=1 and d=0, we have 1≤q≤n−1, and by Lemma 2(ii) it holds that $h(n-q,\Delta ,0_{\leq },\Delta_{\leq })=0$ (resp., $h(n-q,\Delta ,0_{\leq },\Delta_{\leq })=1$), if 1≤q≤n−2 (resp., otherwise (if q=n−1)). This implies that any tree $H\in H(n,\Delta ,1_{=},0_{=})$ has exactly q=n−1 subtrees $H_{v}\in H\left(1,0,0_{\leq },0_{\leq }\right)$, for $v\in N_{H}\left(r_{H}\right)$. However, observe that for each integer m≥2 or d≥1, and *q* satisfying the conditions given in the lemma, there exists at least one tree $H\in H(n,\Delta ,m_{=},d_{=})$ such that *H* has exactly *q* subtrees $H_{v}\in H\left(m,d,m-1_{\leq },d_{\leq }\right)$, for $v\in N_{H}\left(r_{H}\right)$. Hence, this and case (a) (resp., case (b)) imply Lemma 4(i) (resp., Lemma 4(ii)).

Furthermore, it holds that

$$\begin{array}{cc} c(m,d;q)= & \;\frac{(h\left(m,d,m-1_{\leq },d\right)+q-1)!}{(h\left(m,d,m-1_{\leq },d\right)-1)!q!}\\ = & \;\frac{(h\left(m,d,m-1_{\leq },d\right)+q-2)!}{\left(h\left(m,d,m-1_{\leq },d\right)-1\right)!\left(q-1\right)!}\times \frac{\left(h\left(m,d,m-1_{\leq },d\right)+q-1\right)}{q}\\ = & \;c\left(m,d;q-1\right)\times \frac{\left(h\left(m,d,m-1_{\leq },d_{\leq }\right)+q-1\right)}{q}. \end{array}$$

Hence, Lemma 4(iii) and (iv) follow from Lemma 4(i) and (ii), respectively. □

We design a DP algorithm to compute h(n,Δ) based on the recursive structures of $h(n,\Delta ,m_{\leq },d_{\leq })$, $h(n,\Delta ,m_{=},d_{\leq })$ and $h(n,\Delta ,m_{=},d_{=})$, 0≤m≤n−1 and 0≤d≤Δ, as given in Lemmas 1 and 4, where $h\left(n,\Delta \right)=h\left(n,\Delta ,n-1_{\leq },\Delta_{\leq }\right)$ for n≥1 and Δ≥0.

**Lemma** **5.** *For any four integers n−1≥m≥0, and Δ≥d≥0, $h(n,\Delta ,m_{\leq },d_{\leq })$ can be obtained in O(nm(n+Δ(n+d·min{n,Δ}))) time and O(nm(Δ(d+1)+1)) space.*

The proof of Lemma 5 follows from Algorithm 1 and Lemma 6.

**Corollary** **1.** *For any two integers n≥1 and Δ≥0,$h(n,\Delta ,n-1_{\leq },\Delta_{\leq })$ can be obtained in $O(n^{2}\left(n+\Delta \left(n+\Delta \cdot \min\left\{n,\Delta \right\}\right)\right))$ time and $O(n^{2}\left(\Delta^{2}+1\right))$ space.*

Next, for any four integers n−1≥m≥0, and Δ≥d≥0, we present Algorithm 1 for solving the problem of calculating $h(n,\Delta ,m_{\leq },d_{\leq })$. In this algorithm, for each integers 1≤i≤n,0≤j≤Δ,0≤k≤min{i,m}, and 0≤p≤min{j,d}, the variables $h\left[i,j,k_{\leq },p_{\leq }\right]$, $h\left[i,j,k_{=},p_{\leq }\right]$, and $h\left[i,j,k_{=},p_{=}\right]$ store the values of $h\left(i,j,k_{\leq },p_{\leq }\right),h\left(i,j,k_{=},p_{\leq }\right),$ and $h(i,j,k_{=},p_{=})$, respectively.

**Lemma** **6.** *For any four integers n−1≥m≥0, and Δ≥d≥0, Algorithm 1 outputs $h(n,\Delta ,m_{\leq },d_{\leq })$ in O(nm(n+Δ(n+d·min{n,Δ}))) time and O(nm(Δ(d+1)+1)) space.*

**Proof.**  Correctness: For each integer 1≤i≤n,0≤j≤Δ,0≤k≤min{i,m}, and 0≤p≤min{j,d}, all the substitutions and if-conditions in Algorithm 1 follow from Lemmas 1, 2, 3 and 4. Furthermore, the values $h[i,j,k_{\leq },p_{\leq }]$, $h[i,j,k_{=},p_{\leq }]$, and $h[i,j,k_{=},p_{=}]$ are computed by the recursive relations given in Lemmas 1 and 4. This implies that Algorithm 1 correctly computes the required value $h[n,\Delta ,m_{\leq },d_{\leq }]$.

Complexity analysis: There are three nested loops over the variables i,j, and *p* at line 4, which take O(n(Δ(d+1)+1)) time. Following there are five nested loops: over variables i,j,k,p, and *q* at lines 5, 6, 7, 8, and 31, respectively. The loop at line 5 is of size O(n), while the loop at line 6 is of size O(Δ). Similarly, the loops at lines 7 and 8 are of size O(m) and O(d), respectively. The fifth nested loop at line 18 is of size O(n) (resp., O(min{n,Δ})) if p=0 (resp., otherwise). Thus from line 5–36, Algorithm 1 takes $O(n^{2}m)$ (resp., O(nmΔ(n+d·min{n,Δ}))) time if Δ=0 (resp., otherwise). Therefore, Algorithm 1 takes O(nm(n+Δ(n+d·min{n,Δ}))) time.

The algorithm stores three four-dimensional arrays. When Δ=0, for each integer 1≤i≤n, and 1≤k≤min{i,m} we store $h\left[i,0,k_{\leq },0_{\leq }\right],h\left[i,0,k_{=},0_{\leq }\right]$ and $h[i,0,k_{=},0_{=}]$, taking O(nm) space. When Δ≥1, then for each integer 1≤i≤n, 0≤j≤Δ, 1≤k≤min{i,m} and 0≤p≤min{j,d} we store $h\left[i,j,k_{\leq },p_{\leq }\right],h\left[i,j,k_{=},p_{\leq }\right]$ and $h[i,j,k_{=},p_{=}]$, taking O(nmΔ(d+1)) space. Hence, Algorithm 1 takes O(nm(Δ(d+1)+1)) space. □

**Algorithm 1** DP based counting algorithm for $h(n,\Delta ,m_{\leq },d_{\leq })$**Input:** Integers n−1≥m≥0 and Δ≥d≥0.**Output:**$h(n,\Delta ,m_{\leq },d_{\leq })$. $h\left[1,j,0_{=},0_{=}\right]:=h\left[1,j,0_{=},p_{\leq }\right]:=h\left[1,j,0_{\leq },p_{\leq }\right]:=1$; $h\left[i,j,0_{=},p_{\leq }\right]:=h\left[i,j,0_{\leq },p_{\leq }\right]:=0$; $h[2,j,1_{=},p_{=}]:=1;$
$h\left[2,j,1_{=},p_{\leq }\right]:=h\left[2,j,1_{\leq },p_{\leq }\right]:=p+1$ for each 2≤i≤n,0≤j≤Δ, 0≤p≤min{j,d}; **for**
i:=3,4,…,n
**do**   **for**
j:=0,1,…,Δ
**do**     **for**
k:=1,2,…,min{i,m}
**do**       **for**
p:=0,1,…,min{j,d}
**do**         **if**
p=0 and k=1
**then**           $h\left[i,j,1_{=},0_{=}\right]:=h\left[i,j,1_{=},0_{\leq }\right]:=h\left[i,j,1_{\leq },0_{\leq }\right]:=1$         **else** /* p≥1 or k≥2 */           c:=1; $h[i,j,k_{=},p_{=}]:=0;$ /* Initialization */           **if**
p=0
**then**             ℓ:=⌊(i−1)/k⌋           **else** /* p≥1 */             ℓ:=min{⌊(i−1)/k⌋,⌊j/p⌋}           **end if**;           **for**
q:=1,2,…,ℓ
**do**             $c:=c\cdot (h\left[k,p,k-1_{\leq },p_{\leq }\right]+q-1)/q;$             **if**
p=0
**then**               $h\left[i,j,k_{=},p_{=}\right]:=h\left[i,j,k_{=},p_{=}\right]+c\cdot h\left[i-qk,j,\min{\left\{i-kq-1,k-1\right\}}_{\leq },j_{\leq }\right]$             **else** /* p≥1 */               $h\left[i,j,k_{=},p_{=}\right]:=h\left[i,j,k_{=},p_{=}\right]+c\cdot h\left[i-kq,j-pq,k_{=},\min{\left\{j-pq,p-\;1\right\}}_{\leq }\right]+\;\;\;\;h\left[i-kq,j-pq,\min{\left\{i-kq-1,k-1\right\}}_{\leq },j-pq_{\leq }\right]$             **end if**           **end for**;           **if**
p=0**then** /* k≥2 */             $h\left[i,j,k_{=},0_{\leq }\right]:=h\left[i,j,k_{=},0_{=}\right]$           **else** /* p≥1 */             $h\left[i,j,k_{=},p_{\leq }\right]:=h\left[i,j,k_{=},p-1_{\leq }\right]+h\left[i,j,k_{=},p_{=}\right]$          **end if**;          $h\left[i,j,k_{\leq },p_{\leq }\right]:=h\left[i,j,k-1_{\leq },p_{\leq }\right]+h\left[i,j,k_{=},p_{\leq }\right]$        **end if**      **end for**    **end for**  **end for** **end for**; **output**
$h[n,\Delta ,m_{\leq },d_{\leq }]$ as $h(n,\Delta ,m_{\leq },d_{\leq })$.

**Theorem** **1.** *For any two integers n≥1 and Δ≥0, the number of non-isomorphic trees with n vertices and* Δ *self-loops can be obtained in $O(n^{2}\left(n+\Delta \left(n+\Delta \cdot \min\left\{n,\Delta \right\}\right)\right))$ time and $O(n^{2}\left(\Delta^{2}+1\right))$ space.*

**Proof.**  By Jordan [18], we can uniquely consider any tree as a rooted tree by either regarding its unicentroid as the root, or in the case of a bicentroid, by introducing a virtual vertex on the bicentroid and assuming the virtual vertex as the root of the tree. By the definition of a unicentroid, the number of mutually non-isomorphic trees with *n* vertices, Δ self-loops and a unicentroid is $h(n,\Delta ,{\left\lfloor \left(n-1\right)/2\right\rfloor }_{\leq },\Delta_{\leq })$. Further, if *n* is even, then there exist trees with *n* vertices and a bicentroid. This implies that the number of mutually non-isomorphic trees with *n* vertices and Δ self-loops is $h(n,\Delta ,{\left\lfloor \left(n-1\right)/2\right\rfloor }_{\leq },\Delta_{\leq })$ when *n* is odd. Let *n* be an even integer. Then any tree *H* with *n* vertices, Δ self-loops and a bicentroid has two connected components, *A* and *B* obtained by the removal of the bicentroid such that $A\in H(n/2,i,n/2-1_{\leq },i_{\leq })$ and $B\in H(n/2,\Delta -i,n/2-1_{\leq },\Delta -i_{\leq })$ for some 0≤i≤⌊Δ/2⌋, where if Δ is even then for i=Δ/2, both of the components *A* and *B* belong to $H(n/2,\Delta /2,n/2-1_{\leq },\Delta /2_{\leq })$.

Note that for any 0≤i≤⌊(Δ−1)/2⌋, it holds that

$$H\left(n/2,i,n/2-1_{\leq },i_{\leq }\right)\cap H\left(n/2,\Delta -i,n/2-1_{\leq },\Delta -i_{\leq }\right)=\emptyset .$$

Therefore, when Δ is odd (resp., even), the number of mutually non-isomorphic trees with *n* vertices, Δ self-loops, and a bicentroid is

∑i=0⌊(Δ−1)/2⌋h(n/2,i,n/2−1≤,i≤)h(n/2,Δ−i,n/2−1≤,Δ−i≤)+αh(n/2,Δ/2,n/2−1≤,Δ/2≤)+12,

such that α=0 (resp., α=1). Thus, the number of mutually non-isomorphic trees with *n* vertices and Δ self-loops is

(6) h(n,Δ,⌊(n−1)/2⌋≤,Δ≤)+∑i=0⌊(Δ−1)/2⌋h(n/2,i,n/2−1≤,i≤)h(n/2,Δ−i,n/2−1≤,Δ−i≤)+αh(n/2,Δ/2,n/2−1≤,Δ/2≤)+12

such that α=0 (resp., α=1) when Δ is odd (resp., even). Moreover, for each 0≤i≤Δ, Algorithm 1 also computes and stores $h(n/2,i,n/2-1_{\leq },i_{\leq })$ during the calculation of $h(n,\Delta ,{\left\lfloor \left(n-1\right)/2\right\rfloor }_{\leq },\Delta_{\leq })$, and therefore the required result follows from Lemma 6. □

We implemented the proposed DP algorithm and counting trees with a given number of vertices and self-loops. The experimental results in Table 1 show that the proposed method efficiently counts trees with *n* vertices and Δ self-loops.

We next give a lower bound and an upper bound on the number of tree-like polymer topologies with self-loops of a given rank. For this we prove the following results.

**Lemma** **7.** *For an integer n≥2, there exists at least one tree-like polymer with n vertices and* Δ *self-loops if $\Delta \geq \left\lceil \frac{n}{2}\right\rceil +1$.*

**Proof.**  Consider a tree *T* of *n* vertices of diameter $\left\lfloor \frac{n}{2}\right\rfloor$ such that *T* contains a path of length $\left\lfloor \frac{n}{2}\right\rfloor$, in which each non-end vertex has degree at least 3. Observe that when *n* is even, the tree *T* has exactly $\left\lceil \frac{n}{2}\right\rceil -1$ vertices of degree 3, and hence $n-\left\lceil \frac{n}{2}\right\rceil +1=\left\lceil \frac{n}{2}\right\rceil +1$ vertices of degree less than 3. When *n* is odd, the tree *T* has $\left\lceil \frac{n}{2}\right\rceil -3$ vertices of degree 3 and one vertex of degree 4. Thus, in this case, the number of vertices of degree less than 3 is $n-\left\lceil \frac{n}{2}\right\rceil +2=\left(2\left\lceil \frac{n}{2}\right\rceil -1\right)-\left\lceil \frac{n}{2}\right\rceil +2=\left\lceil \frac{n}{2}\right\rceil +1.$ This implies that *T* can be transformed into a polymer with $\left\lceil \frac{n}{2}\right\rceil +1$ self-loops by assigning a self-loop to each vertex of degree less than 3. Hence, $\left\lceil \frac{n}{2}\right\rceil +1$ self-loops are sufficient to get a tree-like polymer with *n* vertices. □

For two integers n≥1 and Δ≥0, let t(n,Δ) denote the number of trees with *n* vertices and Δ self-loops. For r≥1, let p(r) denote the number of tree-like polymers with self-loops and no multi-edges of rank *r*. Observe that a tree with *n* vertices and *k* self-loops at each vertex is a polymer with *n* vertices of cycle rank kn. From this fact and Lemma 7 it holds that

$$\sum_{n,k\in Z^{+}:nk=r}t\left(n,0\right)\leq p\left(r\right)\leq \sum_{n\in Z^{+}:\left\lceil \frac{n}{2}\right\rceil +1\leq r}t\left(n,r\right).$$

## 4. Conclusions

This paper presented an efficient method to count the number of all mutually non-isomorphic trees with a given number of vertices and self-loops. The proposed method is based on dynamic programming where we count the number of all mutually non-isomorphic rooted trees with a given number *n* of vertices and Δ self-loops in $O(n^{2}\left(n+\Delta \left(n+\Delta \cdot \min\left\{n,\Delta \right\}\right)\right))$ time and $O(n^{2}\left(\Delta^{2}+1\right))$ space. As an application of our results, we gave lower and upper bounds on the number of tree-like polymer topologies with a given cycle rank. This is an interesting application of DP to objects such as trees, and offers the advantage of getting the size of the entire solution space at low computational complexity without explicitly generating each object.

An interesting direction for future research is to efficiently generate all mutually non-isomorphic trees with a given number of vertices and self-loops by using the result from the developed counting method. Further, another possible extension of this research is to count and generate all mutually non-isomorphic tree-like polymer topologies with a given number of vertices and self-loops.

## Figures and Tables

**Table 1:** Experimental result of the counting method.

(n,Δ)	Number of Trees	Time [s]
(10,0)	106	0.000173
(20,0)	823,065	0.00048
(10,5)	91,037	0.001193
(10,30)	6,629,790,712	0.00881
(20,10)	5,143,681,226,004	0.006869
(30,10)	2,547,562,522,909,694,331	0.015901
